# Natural killer cell dysfunction is a distinguishing feature of systemic onset juvenile rheumatoid arthritis and macrophage activation syndrome

**DOI:** 10.1186/ar1453

**Published:** 2004-11-10

**Authors:** Joyce Villanueva, Susan Lee, Edward H Giannini, Thomas B Graham, Murray H Passo, Alexandra Filipovich, Alexei A Grom

**Affiliations:** 1 Division of Hematology/Oncology, Children's Hospital Medical Center, Cincinnati, Ohio, USA; 2 William S Rowe Division of Rheumatology, Children's Hospital Medical Center, Cincinnati, Ohio, USA

**Keywords:** juvenile rheumatoid arthritis, macrophage activation syndrome, natural killer cells, perforin, reactive hemophagocytic lymphohistiocytosis

## Abstract

Macrophage activation syndrome (MAS) has been reported in association with many rheumatic diseases, most commonly in systemic juvenile rheumatoid arthritis (sJRA). Clinically, MAS is similar to hemophagocytic lymphohistiocytosis (HLH), a genetic disorder with absent or depressed natural killer (NK) function. We have previously reported that, as in HLH, patients with MAS have profoundly decreased NK activity, suggesting that this abnormality might be relevant to the pathogenesis of the syndrome. Here we examined the extent of NK dysfunction across the spectrum of diseases that comprise juvenile rheumatoid arthritis (JRA). Peripheral blood mononuclear cells (PBMC) were collected from patients with pauciarticular (*n *= 4), polyarticular (*n *= 16), and systemic (*n *= 20) forms of JRA. NK cytolytic activity was measured after co-incubation of PBMC with the NK-sensitive K562 cell line. NK cells (CD56^+^/T cell receptor [TCR]-αβ^-^), NK T cells (CD56^+^/TCR-αβ^+^), and CD8^+ ^T cells were also assessed for perforin and granzyme B expression by flow cytometry. Overall, NK cytolytic activity was significantly lower in patients with sJRA than in other JRA patients and controls. In a subgroup of patients with predominantly sJRA, NK cell activity was profoundly decreased: in 10 of 20 patients with sJRA and in only 1 of 20 patients with other JRA, levels of NK activity were below two standard deviations of pediatric controls (*P *= 0.002). Some decrease in perforin expression in NK cells and cytotoxic T lymphocytes was seen in patients within each of the JRA groups with no statistically significant differences. There was a profound decrease in the proportion of circulating CD56^bright ^NK cells in three sJRA patients, a pattern similar to that previously observed in MAS and HLH. In conclusion, a subgroup of patients with JRA who have not yet had an episode of MAS showed decreased NK function and an absence of circulating CD56^bright ^population, similar to the abnormalities observed in patients with MAS and HLH. This phenomenon was particularly common in the systemic form of JRA, a clinical entity strongly associated with MAS.

## Introduction

The term 'macrophage activation syndrome' (MAS) in pediatric rheumatology refers to a set of symptoms caused by the excessive activation and proliferation of T cells and well-differentiated macrophages [1-4]. This activation leads to an overwhelming inflammatory reaction that can be fatal. The pathognomonic features of this syndrome are found in bone marrow aspirates: numerous, well-differentiated macrophages (or histiocytes) actively phagocytosing hematopoietic elements. Although MAS has been increasingly recognized in association with almost any rheumatic disease, it is by far most common in the systemic form of juvenile rheumatoid arthritis (JRA) [1,5-11].

Clinically, MAS has strong similarities to familial hemophagocytic lymphohistiocytosis (FHLH) and virus-associated or reactive hemophagocytic lymphohistiocytosis (HLH) [2-4]. The immune abnormalities in the familial form of HLH have been studied extensively, and the most consistent finding has been global impairment of cytotoxic lymphocyte and natural killer (NK) cell function [12-14]. In about 50% of patients with FHLH in North America, these immunologic abnormalities are secondary to mutations in the gene encoding perforin, a protein that mediates the cytotoxic activity of NK and T cells [15]. Although it has been proposed that abnormal cytotoxic cells might fail to provide appropriate apoptotic signals for the removal of activated macrophages and T-cells after infection is cleared [16], the exact pathways that would link the decreased NK and cytotoxic T cell function with macrophage expansion have not been confirmed.

We have previously reported that, as in HLH, NK function is profoundly depressed in the vast majority of patients with MAS [17] suggesting that this immunologic abnormality might be relevant to the pathogenesis of the syndrome. In the present study we sought to assess the extent of NK dysfunction in the most common rheumatic disease of childhood, JRA.

JRA is a chronic, idiopathic, inflammatory disorder with diverse clinical symptoms both at onset and during the course of the disease. Classification of this heterogeneous disease has been based primarily on the type of onset, namely the clinical manifestations during the first six months [18,19]. There are at least three major onset types: pauciarticular (four or fewer joints involved), polyarticular (five or more joints), and systemic. The systemic onset form, with its markedly febrile presentation, is certainly the most distinct clinical subtype of the disease. In contrast to patients with pauciarticular and polyarticular JRA, in whom the joint disease usually overshadows the more general symptomatology, in systemic onset JRA extra-articular features such as spiking fevers, evanescent macular rash, hepatosplenomegaly, lymphadenopathy, and, occasionally, polyserositis are most prominent [20]. The reason for the increased incidence of MAS in patients with systemic forms of JRA in comparison with other clinical forms of this disease is not clear, but NK cell abnormalities might have a role [3]. The main purpose of this cross-sectional study was to characterize numbers of circulating NK cells, their cytolytic activity, CD56^bright ^: CD56^dim ^subset ratio, and perforin/granzyme B expression in the major cytotoxic cell populations in patients with different clinical forms of JRA.

## Materials and methods

### Patients

In all patients included in the study, the diagnosis of JRA was established on the basis of the American College of Rheumatology (ACR) diagnostic criteria [18]. The main clinical characteristics of the patients are summarized in Table 1. Peripheral blood samples were collected from the patients after obtaining informed consent under an institutional review board-approved study of JRA.

### Flow cytometric analysis

Relative and absolute numbers of NK, CD8^+^, and NK T cells, as well as perforin and granzyme B expression in these cell populations, were determined as described previously [14,21]. In brief, whole blood samples were first surface stained with the following antibodies: fluorescein isothiocyanate-labelled T cell receptor (TCR)-αβ, CD8-peridinin chlorophyll protein (CD8-PerCP) (BD Immunocytometry Systems, San Jose, CA), and CD56-allophycocyanin (CD56-APC) (Immunotech, Brea, CA). Red cells were then lysed with FACSlyse (BD Immunocytometry Systems) and washed. The resultant white cell pellets were then fixed and permeabilized with Cytofix/Cytoperm (BD Pharmingen, San Diego, CA) and stained with either phycoerythrin-conjugated mouse IgG2b anti-perforin or phycoerythrin-conjugated mouse IgG1 anti-granzyme B antibodies (BD Pharmingen). After being washed, cells were resuspended in 1% paraformaldehyde and stored at 4°C until analyzed with a FACSCalibur flow cytometer (Becton Dickinson, San Jose, CA). The following gates were used to distinguish the three populations of interest: CD8^+ ^T cells were defined as being TCR-αβ^+^, CD8^+^, and CD56^-^; NK cells as TCR-αβ^- ^and CD56^+^; and NK T cells as TCR-αβ^+ ^and CD56^+^. All populations were also restricted to a live cell gate based on forward versus side scatter. The perforin-positive or granzyme B-positive regions were set by using isotype-matched negative control samples, and the percentage positive for each gate was reported.

### NK cell cytotoxicity analysis

NK activity was assessed after co-incubation of peripheral blood mononuclear cell preparations (effector cells) with ^51^Cr-labeled target cells at various effector : target cell ratios as described previously [21]. The NK-sensitive K562 line was used as a source of target cells. The levels of radioactivity released from target cells into supernatants were assessed by gamma scintillation after 4 hours of incubation. All experiments were performed in triplicate in a 96-well microtiter plate. Spontaneous and maximum release wells were included on each plate as controls. Spontaneous release was assessed in the wells containing ^51^Cr-labeled target cells in medium without effector cells. Maximum release was determined in the wells containing labeled target cells in the presence of detergent to promote total lysis. The percentage lysis was calculated as described previously [21]: percentage lysis = 100 × (mean radioactivity of sample minus mean radioactivity of background)/(mean maximum radioactivity minus mean radioactivity of background).

Lytic units were calculated from the curve of the percentage lysis. One lytic unit was defined as the number of effector cells needed to produce 10% lysis of 10^3 ^target cells during the 4 hours of incubation.

### Controls

The results were compared with the normal ranges for age-matched controls that have been developed in our clinical laboratory by studying 41 pediatric samples obtained from the out-patient clinic during routine 'well-child' visits from children considered 'healthy' [14].

### Statistical analysis

The unpaired *t*-test and Wilcoxon two-sample test were used to compare NK cytolytic activity and perforin/granzyme B expression between the patient and control groups. The rank correlation test was used to characterize the relationship between NK cell activity and perforin expression. The unpaired *t*-test and logistic regression analysis were used to assess the possible contribution of treatment regimens to the development of NK cell dysfunction.

## Results

### NK cell cytolytic activity and NK cell numbers

**Figure 1:**
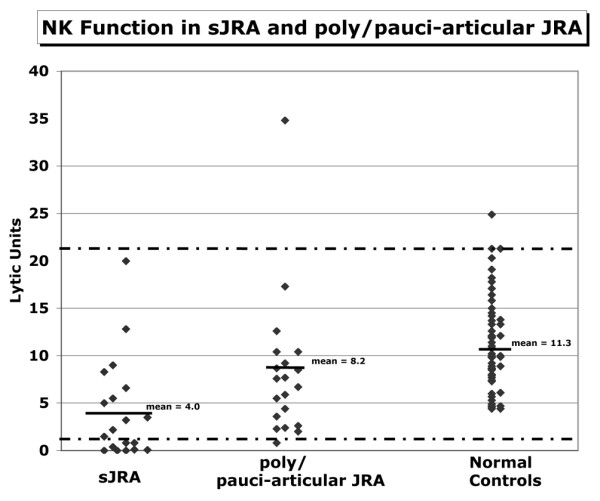
NK cell cytolytic activity in patients with juvenile rheumatoid arthritis (JRA). Activity of NK cells was determined after co-incubation of peripheral blood mononuclear cells with the NK-sensitive K562 cell line and expressed in cytolytic units (LU, *y*-axis). NK activity values in 20 patients with systemic JRA, 20 patients with other subtypes of JRA and 41 healthy children are shown as dots. The difference between results for patients with systemic JRA and other JRA is statistically significant (Wilcoxon two-sample test, *P *= 0.0062). For comparison, mean ± 2SD values determined in healthy individuals are shown as horizontal lines.

As shown in Fig. 1, some decrease in NK cell cytolytic activity was noted in both clinical groups of JRA patients. This trend was particularly strong in patients with systemic JRA (sJRA). The mean cytolytic activity in the sJRA group was 4.0 (SEM = 1.2) compared with 8.2 (SEM = 1.6) in patients with pauciarticular/polyarticular JRA (unpaired *t*-test, *P *= 0.042; Wilcoxon two-sample test, *P *= 0.0062). Furthermore, in a subgroup of patients with sJRA, NK cell function was profoundly depressed. Thus, in 10 of 20 patients with sJRA and in only 1 of 20 patients with pauciarticular/polyarticular JRA, the NK cell cytolytic activity was below two standard deviations (SD) of the control group (χ^2^, *P *= 0.002). The same degree of NK cell dysfunction was observed in our previous studies of patients with MAS and HLH [17].

As shown in Table 1, a significant proportion of the patients studied were on immunosuppressive medications, including prednisone, methotrexate, and tumor necrosis factor (TNF)-blocking agents. Because NK function can be affected by such medications [22], we sought to assess whether the differences in treatment regimens between patients with sJRA and pauciarticular/polyarticular JRA might have contributed to the observed differences in NK function. Overall, patients receiving immunosuppressive drugs had somewhat lower levels of NK cytolytic activity. However, logistic regression analysis showed no statistically significant differences in NK function between patients with or without immunosuppressive treatment (independent variables: prednisone, methotrexate, and TNF-blocking agents; dependent variable NK cytolytic activity below 2SD of control samples, χ^2 ^= 0.877, *P *= 0.831).

Because low NK cell numbers in patients with sJRA have been previously noted in one study [23], we assessed whether the decrease in NK cell cytolytic activity might have been related to low NK cell counts. A moderate correlation between NK function and the proportion of peripheral blood mononuclear cells that were NK cells was found for both groups of patients with JRA (*r *= 0.52, 95% confidence interval 0.08–0.8 in the sJRA group; *r *= 0.47, 95% confidence interval 0.7–0.75 in the other JRA group). Correlation coefficients between function and number of NK cells were not significantly different between the two JRA groups (Fisher's *Z *transformation; *P *= 0.7). The mean number of NK cells (expressed as a proportion of TCR^-^αβ^-^/CD56^+ ^cells in a population of peripheral blood mononuclear cells) among the sJRA group was 0.077 (SD 0.04) in comparison with 0.081 (SD 0.034) among the other JRA group was not significantly different (*P *= 0.72 on the basis of the two-tailed independent *t*-test). Thus, it seems that suppressed NK function is not simply a result of reduced numbers of NK cells among patients with sJRA.

### CD56^dim ^and CD56^bright ^NK cells

**Figure 2:**
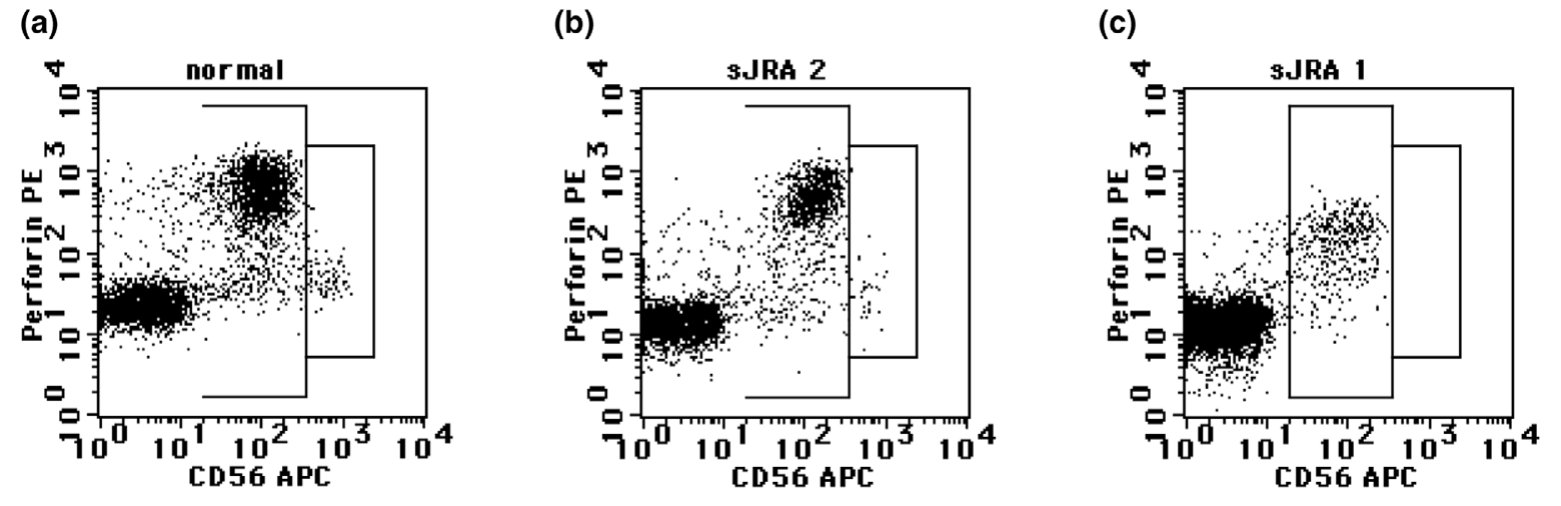
Flow cytometric analysis of CD56^bright ^and CD56^dim ^natural killer cells. (**a**) In healthy individuals, most circulating NK cells are CD56^dim ^(about 90%) and express high levels of perforin. In contrast, CD56^bright ^NK cells comprise about 10% of all human NK cells and express low levels of perforin. (**b, c**) Two distinct patterns of CD56 staining observed in patients with systemic juvenile rheumatoid arthritis (sJRA): (b) normal pattern (observed in 17 of 20 patients with sJRA as well as in all controls, and all other JRA patients); (c) almost complete disappearance of CD56^bright ^subset of NK cells (observed in 3 of 20 patients with sJRA only).

On the basis of on the intensity of CD56 staining, human NK cells have been recently subdivided into two distinct subsets with distinct functional characteristics. CD56^bright ^NK cells have the ability to produce high levels of immunoregulatory cytokines, in particular interferon (IFN)-γ, but are in general poorly cytotoxic (reviewed in [24]). By contrast, CD56^dim ^NK cells produce relatively low levels of cytokines and are potent cytotoxic effector cells expressing high levels of peforin. We have previously described a lack of the circulating CD56^bright ^NK cells in patients with MAS and HLH [25]. The analysis of the fluorescence-activated cell sorting data in the current study revealed that three patients with sJRA had a similar abnormality, namely an almost complete absence of circulating CD56^bright ^cells. Figure 2 shows examples of CD56 staining in such patients.

### Perforin expression

**Figure 3:**
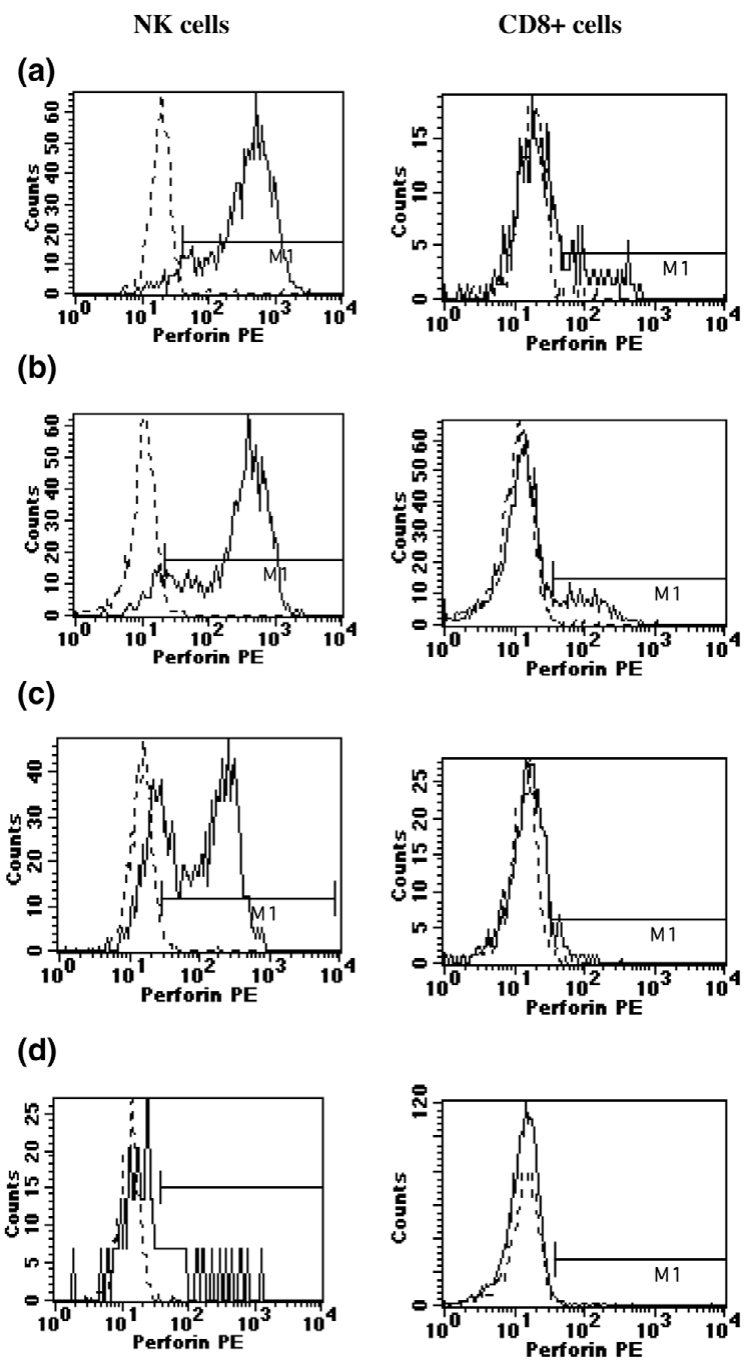
Flow cytometric analysis of perforin expression in NK cells and cytotoxic CD8^+ ^cells. (**a**) Normal control: 95% of NK cells and 14% of CD8^+ ^cells are perforin-positive. (**b**) A patient with systemic juvenile rheumatoid arthritis (sJRA) with a normal pattern of perforin expression: normal mean channel fluorescence (MCF) in NK cells with 92% of NK cells and 10% of CD8^+ ^T cells being perforin-positive (this pattern was observed in all controls, in 18 of 20 other JRA patients, and in 15 of 20 sJRA patients). (**c**) A patient with sJRA with low MCF in NK cells and mildly decreased proportions of perforin-positive NK cells (74%) and CD8^+ ^T lymphocytes (5%) (this pattern was observed in 2 of 20 other JRA patients and in 5 of 20 sJRA patients). (**d**) A patient with sJRA with low MCF in NK cells and very low proportions of perforin-positive NK cells (20%) and no perforin-positive CD8^+ ^T lymphocytes (observed in 1 of 20 sJRA patients only).

Because reduced perforin expression in cytotoxic effector cells has previously been reported in sJRA [26,27], we assessed perforin content and relative proportions of perforin-positive NK cells, CD8^+ ^lymphocytes, and NK T cells. High variability was noted in both groups of patients with JRA. The comparison of the mean values between patients with sJRA versus other JRA groups did not reveal statistically significant differences. However, the examination of individual patterns of perforin staining in NK cells revealed mean channel fluorescence (MCF) values below 2SD of the control group in seven patients, five of whom had sJRA. In addition to low MCF, one of these patients with sJRA had profoundly decreased proportions of perforin-positive cells in all three cytotoxic cell populations, a pattern that we have previously described in patients with sJRA who have had multiple episodes of MAS [17]. Examples of such abnormal patterns of perforin expression are shown in Fig. 3. In contrast, the patterns of staining for granzyme B were similar between patients and controls in all three cytotoxic cell populations (data not shown).

Because perforin is a protein that mediates the cytotoxic activity of NK cells, we assessed whether decreased perforin expression might have contributed to the development of NK dysfunction in JRA. In the group of seven patients with low perforin levels in NK cells, only three had NK cytolytic activity below 2SD of the control group. Furthermore, there was no significant correlation between MCF in NK cells and their cytolytic activity (*r *= 0.1596 for patients with sJRA, and *r *= 0.1991 for patients with other JRA subtypes).

## Discussion

In this study, profoundly depressed NK cell activity was observed in a large subgroup of patients with sJRA and in only 1 of 20 patients with the polyarticular form of the disease. The extent of NK dysfunction in this group of patients was similar to that seen in patients with MAS [17] or HLH [12,13]. The two study groups (sJRA versus other JRA subtypes) were well matched in terms of age, duration of the disease, and treatment regimens with the exception of a slightly higher proportion of patients with sJRA receiving steroids. Steroids have been reported to suppress the cytolytic activity of NK cells [22], and this might potentially have contributed to the observed differences in NK function. However, the logistic regression analysis did not show significant differences between groups defined on the basis of treatment regimens. In addition, several patients with sJRA who demonstrated profoundly depressed NK cell cytolytic activity were receiving only non-steroidal anti-inflammatory drugs. Owing to the limitations of the statistical power with the numbers of study subjects enrolled, it is possible that some effects of the immunosuppressive medications might have been underestimated. Nevertheless, NK dysfunction seems to be a distinguishing feature of sJRA that is intrinsic to the disease itself.

Further analysis of the flow cytometry data revealed that some of the patients with sJRA had a rather selective disappearance of the circulating immunoregulatory CD56^bright ^subset of NK cells, a pattern previously seen in patients with MAS or HLH [25]. These cells express low levels of perforin and are, in general, poorly cytotoxic [24]. The disappearance of CD56^bright ^NK cells from peripheral circulation is therefore unlikely to account for the defects in cytolytic activity of NK cells. In contrast, CD56^bright ^NK cells might have a function in regulating the CD56^dim ^perforin^bright ^cells, and in this case their disappearance might have an effect on cytolytic activity in some sJRA patients. Alternatively, the apparent absence of immunoregulatory NK cells in peripheral circulation might reflect their active recruitment to sites of inflammation.

Although the observed NK dysfunction in a subgroup of patients with sJRA might not be of primary etiological significance for JRA itself, the similarities to the immunologic abnormalities seen in MAS and HLH suggest that depressed NK cell activity is likely to be relevant to the pathogenesis of MAS in sJRA. It is important to mention that low NK cell activity has been noted in many rheumatic diseases [28], most notably in systemic lupus erythematosus [29]. In our study, however, in a subgroup of patients with sJRA, the extent of NK dysfunction was profound, with an almost complete absence of cytolytic activity. This parallels the fact that, although MAS has been described in association with almost any rheumatic disease and it is not uncommon in systemic lupus erythematosus [30], it is by far most common in sJRA [4-11].

Other groups have noted low levels of perforin expression in cytotoxic cells from patients with sJRA in comparison with other clinical forms of the disease, suggesting that this feature might be responsible for the increased incidence of MAS [26,27]. In our study, when patients with sJRA were analyzed as a group, perforin levels were not significantly different from those in patients with other JRA types. However, the examination of the individual patterns of perforin staining revealed a small subgroup of JRA patients with a very low perforin content in NK cells. Most of these patients had sJRA. Furthermore, one of them had profoundly decreased proportions of perforin-positive cells in all three major cytotoxic cell populations, a pattern that has been previously reported in patients with MAS [17] and in the carriers of perforin-deficient FHLH [14]. Although no overall correlation between perforin expression and NK cell cytolytic activty was noted in our study, we still cannot exclude the possibility that, at least in some patients, reduced perforin expression might have functional significance. In other words, there might be some heterogeneity in the mechanisms underlying NK cell dysfunction in sJRA. The existence of such heterogeneity was also noted in our previous study of MAS patients [17] that included ethnically diverse Caucasian, African American, and Latin American patients. The ethnic heterogeneity of the patients with JRA included in this study might also underlie the discrepancy between our results and the study by Wulffraat and colleagues [26], which showed that patients with sJRA as a group had lower perforin expression in cytotoxic effector lymphocytes. That study included a much more ethnically homogeneous population of Dutch children.

Granzyme B is another important component of the perforin-mediated cytotoxicity pathway. In our study both patient groups had granzyme B expression patterns indistinguishable from those seen in healthy controls, suggesting that the observed NK dysfunction is not likely to be related to abnormal granzyme B expression.

The cytolytic activity of NK cells in our study was measured by using NK-sensitive K562 cells, which are lymphoblasts derived from a patient with chronic myelogenous leukemia. The exact receptors involved in the NK-mediated lysis of K562 cells have not yet been identified. However, the lysis of some similar cell lines has been recently shown to be mediated through the natural cytotoxicity receptors (NKp46, NKp30, and NKp44) [31]. These receptors have important biologic functions in the innate immune system, and their abnormal expression might have a function in the development of NK dysfunction in sJRA.

On the basis of our data, the feature that distinguishes systemic onset JRA from other forms of JRA, and is common to the major hemophagocytic syndromes, is NK cell dysfunction. The exact mechanisms that would link deficient NK cell function and, in some cases, depressed perforin expression with the expansion of activated macrophages are not clear. One possible explanation is that decreased NK function might be responsible for a diminished ability to clear the infecting pathogen and remove the source of antigenic stimulation at early stages of infection [32]. This would lead to persistent antigen-driven T cell activation associated with an increased production of cytokines, such as IFN-γ and granulocyte/macrophage colony-stimulating factor, that stimulate macrophages. Subsequently, the sustained macrophage activation would result in tissue infiltration and in the production of high levels of TNF-α, interleukin-1, and interleukin-6, which have a major role in the various clinical symptoms and tissue damage.

Several recent studies using perforin-deficient and NKcell-depleted mice indicate that NK cells and perforin-based systems are also involved in the downregulation of immune responses through a direct effect of NK cells and/or perforin-based systems on the survival of activated lymphocytes [33-36]. NK dysfunction might therefore lead to a failure to provide homeostatic signals for the removal of activated T cells. For instance, Su and colleagues [33] demonstrated that the infection of NK-depleted mice with murine CMV results in an exaggerated immune response associated with more persistent expansion of cytotoxic CD8^+ ^T cells that secrete IFN-γ, an important macrophage activator. Another possible explanation is related to the recently discovered ability of NK cells to lyse autologous antigen-presenting cells such as dendritic cells, thus limiting the magnitude of an immune response [37]. Interestingly, this interaction might involve the above-mentioned natural cytotoxicity receptors [38].

## Conclusions

NK cell dysfunction is the feature that distinguishes systemic onset JRA from other forms of JRA, and is common to the major hemophagocytic syndromes. This suggests that impaired cytotoxic functions and/or deficiency of immunoregulatory NK cells are relevant to the development of MAS. Patients with sJRA who have these immunologic abnormalities may therefore be a high-risk group that might benefit from closer observation.

## Abbreviations

IFN = interferon; HLH = hemophagocytic lymphohistiocytosis; JRA = juvenile rheumatoid arthritis; MAS = macrophage activation syndrome; NK = natural killer; MCF = mean channel fluorescence; sJRA = systemic juvenile rheumatoid arthritis; TCR = T cell receptor; TNF = tumor necrosis factor.

## Competing interests

The author(s) declare that they have no competing interests.

## Authors' contributions

JV carried out sample collection, flow cytometry, data analysis and manuscript preparation. SL carried out NK cytotoxicity assays and manuscript preparation. EHG carried out statistical analysis and manuscript preparation. TBG carried out patient referral, clinical data analysis and manuscript preparation. MHP carried out patient referral, clinical data analysis and manuscript preparation. AF carried out study design, NK studies oversight and manuscript preparation. AAG carried out study design, project oversight, patient referral, data analysis and manuscript preparation. All authors read and approved the final manuscript.

## Figures and Tables

**Table 1:** Clinical characteristics of patients with juvenile rheumatoid arthritis

Parameter	Systemic onset JRA (*n *= 20)	Polyarticular JRA (*n *= 16) or pauciarticular JRA (*n *= 4)
Male/female	9/11	14/6
Mean age, years (range)	11.5 (2–23)	12.8 (3–22)
Mean disease duration, years (range)	4.1 (0.2–16)	5.8 (0.9–15)
No. with active arthritis (%)	17 (85)	17 (85)
No. with active systemic disease (%)	8 (40)	-
Mean ESR, mm/h (range)	30 (5–85)	21 (5–57)
No. (%) receiving		
Prednisone	6 (30)	3 (15)
Methotrexate	12 (60)	10 (50)
TNF-blocking agent	3 (15)	5 (25)
Intra-articular steroids	1 (5)	4 (20)
NSAID	17 (85)	18 (90)

ESR, erythrocyte sedimentation rate; JRA, juvenile rheumatoid arthritis; NSAID, non-steroidal anti-inflammatory drugs; TNF, tumor necrosis factor.
